# Minimally Invasive Sacroiliac Joint Fusion: One-Year Outcomes in 40 Patients

**DOI:** 10.1155/2013/536128

**Published:** 2013-08-13

**Authors:** Donald Sachs, Robyn Capobianco

**Affiliations:** ^1^Center for Spinal Stenosis and Neurologic Care, P.O. Box 8815, Lakeland, FL 33806, USA; ^2^SI-BONE Inc., 3055 Olin Ave. Suite 2200, San Jose, CA 95128, USA

## Abstract

*Background*. SI joint pain is difficult to diagnose due to overlapping symptoms of the lumbar spine, and until recently, treatment options have been limited. The purpose of this retrospective study is to report on the safety and effectiveness of MIS SI joint arthrodesis using a series of triangular, porous plasma coated implants in patients refractory to conservative care. *Methods*. We report on the first 40 consecutive patients with one-year follow-up data that underwent MIS SI joint fusion with the iFuse Implant System (SI-BONE, Inc., San Jose, CA) by a single surgeon. Medical charts were reviewed for demographics, perioperative metrics, complications, pain scores, and satisfaction. *Results*. Mean age was 58 years (range 30–81) and 75% of patients were female. Postoperative complications were minimal and included transient trochanteric bursitis (5%), facet joint pain (20%), and new low back pain (2.5%). There were no reoperations at one year. Mean pain score improved from 8.7 (1.5 SD) at baseline to 0.9 (1.6) at 12 months, a 7.8-point improvement (*P* < .001). Patient satisfaction was very high. *Conclusions*. The results of this case series reveal that MIS SI joint fusion using the iFuse Implant System is a safe and effective treatment option in carefully selected patients.

## 1. Background 

Low back pain (LBP) is exceedingly common in modern society, affecting well over 90% of adults at some point in their lives [1]. Apart from the common cold, it is the most common reason for visits to the primary care doctor [1]. Loss of productivity and income combined with medical expenses results in a $60 billion expenditure annually in the US related to low back pain [2]. Successful treatment of low back pain demands identifying the pain generator(s), which can be a significant challenge due to the multifactorial nature of this condition. In the early 1900s, the sacroiliac (SI) joint was suspected as a significant generator of LBP. Over time, as more reliably diagnosed conditions such as herniated discs and facet arthropathy became better understood, less focus was placed on the SI joint [3]. Recently there has been a resurgence in consideration of the SI joint as a low back pain generator. Recent published literature reports that 15–30% of patients presenting with low back pain had SI joint problems [4]. Additionally, up to 75% of postlumbar fusion patients will develop significant SI joint degeneration after 5 years [5–7]. SI joint pain can mimic discogenic or radicular low back pain, and patients can present with low back, groin, and/or gluteal pain, leading to the potential for inaccurate diagnosis and treatment [1, 8, 9].

Despite the large number of patients with SI joint pain, treatment options have been limited to conservative care involving physical therapy and joint injections or traditional open SI joint arthrodesis surgery until recently. Open arthrodesis procedures reported in the literature require relatively large incisions, significant bone harvesting, and lengthy hospital stays; moreover, they may require nonweight bearing for several months [10–13]. 

Recent case series reports of a minimally invasive arthrodesis system (iFuse Implant System, SI-BONE Inc., San Jose, CA) have shown excellent outcomes [14–17]. The surgical procedure involves placing a series of triangular, porous plasma spray coated titanium implants placed across the SI joint without the use of second site bone harvesting or graft. We wished to determine if the single center outcomes in the literature were commensurate with our own. The purpose of this retrospective study is to report on the safety and effectiveness of this procedure in a single surgeon's private practice.

## 2. Methods

We report outcomes of the first consecutive 40 patients with one-year follow-up data treated at a single, community-based spine practice between April 2011 and March 2012. Medical charts were reviewed for perioperative metrics, complications, and pain scores using a numerical rating scale (NRS) preoperatively and at 6 weeks, 3-, 6-, and 12-months post-operatively. Patient satisfaction with surgical results (yes or no) was obtained at 12 months post-operatively. IRB approval was obtained before beginning the study.

Mean age was 58 years (range 30–81), and three quarters of the patients were women (75%) (Table 1). Patients were diagnosed with either degenerative sacroiliitis or sacroiliac joint disruption using a combination of history, clinical exam, and positive diagnostic injection. Degenerative sacroiliitis is defined as a degeneration of the joint, either osteoarthritic or as a result of adjacent segment disease after fusion. Sacroiliac joint disruption is a physical separation of the joint, typically as a result of trauma. Patients presented with SI joint pain and all but one complained of low back pain. Additional symptoms were buttock pain (60%) and groin pain (13%). Nearly half (48%) had a history of previous lumbar spine surgery that included: fusion at one or more levels (63%), decompression (16%), discectomy (10.5%), and 10.5% with nonspecific documented procedures.

All patients failed a minimum 6-month course of conservative care consisting of medication optimization, physical therapy, and SI joint injections. A thorough physical and clinical exam was performed on all patients in order to determine the primary pain generator as accurately as possible in this complex back pain population. Positive results on 3 or more provocative physical examination maneuvers (such as FABER, compression, thigh thrust, distraction, and Gaenslen) were used as criteria to guide subsequent diagnostic activities [18]. Diagnostic imaging studies (MRI and/or CT scan) were performed to assess pathology in the lumbopelvic hip complex. When clinical, physical, and imaging examinations were concordant, patients were sent for confirmatory image-guided diagnostic injections of the SI joint using long acting anesthetic. A 75% reduction in pain immediately following injection of local anesthetic was used to confirm the SI joint as a pain generator [7]. 

Minimally invasive SI joint fusion using the iFuse Implant System (SI-BONE Inc., San Jose, CA) was performed in all cases by a single neurosurgeon in private practice. This system entails the placement of 3 triangular, porous plasma coated titanium implants across the SI joint in order to stabilize and fuse the joint without the need for additional bone graft. 

### 2.1. Technique Overview

**Figure 1:**
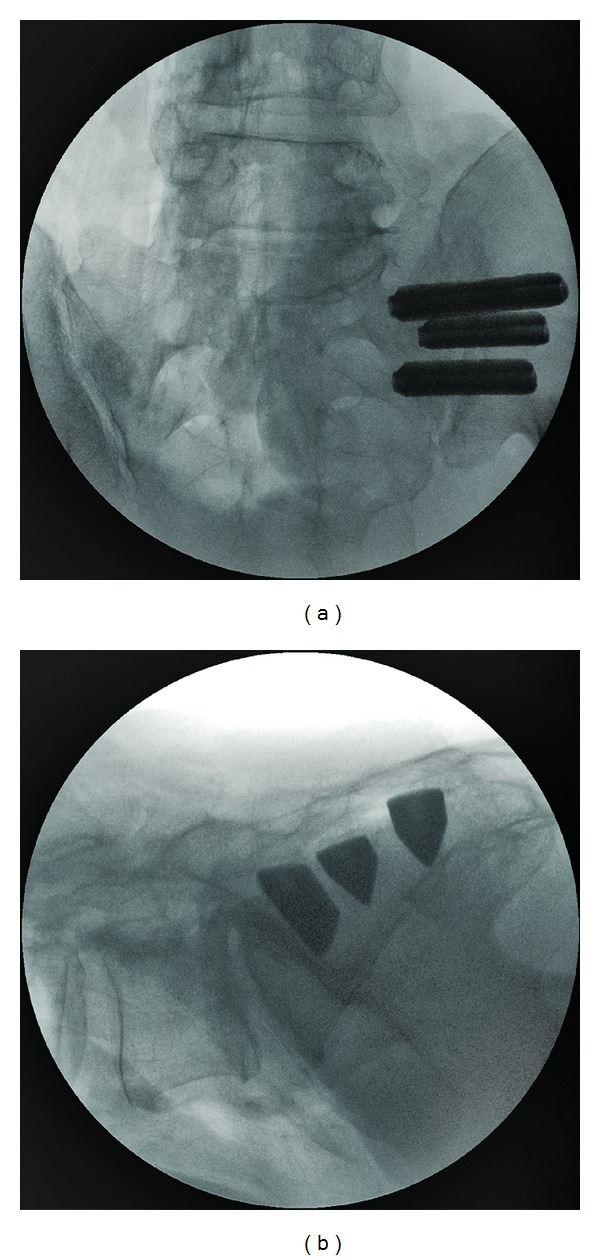
(a) AP and (b) lateral view of all three implants in place.

The procedure is performed with the patient positioned prone on a radiolucent table and under general endotracheal anesthesia. Intermittent fluoroscopy is used to monitor instrument and implant placement. After a 3 cm lateral incision is made into the buttock region, the gluteal fascia is bluntly dissected to reach the outer table of the ilium. A Steinmann pin is passed through the ilium across the SI joint into the center of the sacrum, lateral to the neural foramen. A soft tissue protector is inserted over the pin, and a drill is used to create a pathway and decorticate bone through the ilium to the sacrum. After the drill is removed, a triangular broach is used before the first implant is malleted into place. A total of three implants are placed. (Figure 1). In some cases more than three implants can be used, but in this series all patients had three implants. The most cephalad implant is seated within the sacral ala. A pin-guide system is used to facilitate placement of the subsequent implants. The second implant is generally located above or adjacent to the S1 foramen and the third between the S1 and S2 foramens. The incision is then irrigated, and the tissue layers are closed with Vicryl and Monocryl sutures. Patients are instructed to ambulate with the assistance of a walker for the first 4 weeks after which time toe touch ambulation is recommended for another 4 weeks. After patients have undergone this 8-week program of a gradual return to full weight bearing, they begin 4 weeks of physical therapy.

### 2.2. Outcomes

Pain related to the SI joint was assessed preoperatively and postoperatively at 12 months. Patients were asked to rate their pain using a 0–10 numerical rating scale with 0 representing no pain and 10 representing the worst pain imaginable. Satisfaction was assessed by asking the patient (yes or no) if s/he would have the same surgery again for the same outcome. 

## 3. Results

A total of 41 SI joints in 40 patients were treated: 17 right- and 24 left sided. One patient underwent bilateral surgery (Table 2). One patient underwent concomitant L3/4 laminectomy, foraminotomy, and facetectomy. Blood loss was minimal (<50 cc) in all cases, and most patients are kept in the hospital overnight. Surgery time was not available for all patients. We previously reported an operating time of 78 ± 32 minutes in a subset of this cohort [15]. No intraoperative complications were observed. At one year, there were no surgical revisions. 

### 3.1. Clinical Outcomes

Mean (±SD) preoperative pain score as measured using a numerical rating scale (NRS) was 8.7 ± 1.5. Improvement in pain was observed as early as the 6-week follow-up visit (mean 1.2 ± 1.7), and patients continued to have symptom relief at the 3- and 6-month follow-up visits, means 0.6 ± 1.2 and 0.8 ± 1.8, respectively. This improvement was durable through the 12-month followup with a reported mean pain score of 0.9 (±1.6). The mean (±SD) change in pain score was −7.8 (±2.3) points (*t* test, *P* < .001). A subgroup analysis revealed that there was no difference in outcomes between patients with and without prior lumbar spinal fusion. A clinically significant benefit, defined as a >2 point change from baseline, was observed in all but one patient [19]. Patient satisfaction was extremely high with all patients (100%) indicating that they would have the same surgery again for the same result. 

### 3.2. Complications

There were no intraoperative complications. Two patients presented with trochanteric bursitis, 1 incident of piriformis syndrome, and 1 episode of new low back pain (Table 3). Eight patients continued to have facet pain, which was present preoperatively. During the postoperative follow-up period, 2 patients with preexisting lower back pain due to degenerative disc disease and severe spinal stenosis underwent lumbar fusion. One patient underwent discectomy at L4/5. All three surgeries were unrelated to the index procedure.

## 4. Discussion 

SI joint symptoms can present as pain in the SI joint, low back, hip, groin, or buttock. As a result, a careful and thorough clinical and physical exam must be performed to correctly identify the pain generator(s). Positive provocative physical examination maneuvers (such as FABER, Gaenslen, and Thigh Thrust) combined with marked (e.g., 75% or greater) pain relief after image-guided SI joint injection are a reliable method for diagnosing the SI joint as a pain generator [7, 18, 20].

Recent reports of other MIS approaches to SI joint arthrodesis using screws show relatively good clinical results with room for improvement in outcomes and technique [16]. Al-Khayer et al. reported on 9 patients using a single hollow modular anchorage (HMA) screw packed with bone graft [21]. All patients experienced a clinically significant improvement in VAS pain scores, and all but 1 patient improved in function as measured by ODI. One patient suffered a deep wound infection. Khurana et al. also report on HMA screws with demineralized bone matrix in a cohort of 15 patients with relatively good outcomes [22]. Wise and Dall reported on 13 patients and 19 joints using 11 × 25 mm threaded fusion cages packed with rhBMP-2 with good clinical results [3]. Both of these MIS techniques, which use rhBMP-2 or autologous bone graft, have substantial drawbacks. The use of rhBMP-2 has come under fire for unreported adverse events as well as unapproved uses [23], and autologous iliac crest harvesting can lead to further degeneration of the SI joint [10]. Additionally, the use of cages and screws for SI joint fusion may not be appropriate for patients with a history of instrumented spinal surgery. Mason et al. reported significantly worse outcomes after SI joint fusion using HMA screws in patients with a history of previous lumbar spine surgery [24]. Further studies are needed to assess the incidence of screw loosening, breakage, and need for hardware removal as these events have been reported in association with other spine procedures using orthopedic screws [19]. 

Several case series reports using the same MIS technique used in this current study report favorable results with minimal complications and no suggestion of implant loosening [14, 15, 17]. In a case series of 50 patients, the author reported clinically and statistically significant improvements in pain and function independent of a prior history of lumbar spine fusion [16]. Similarly, there was no difference in outcomes between patients with and without history of lumbar spinal fusion in our study.

Advantages of MIS SI joint fusion using the iFuse Implant System include a small incision, relatively short operating time, minimal blood loss, a relatively short period of immobilization, and most importantly bone and ligament preservation. The triangular shape combined with an interference fit of the titanium implant used in this cohort was designed to minimize rotation, and micromotion and avoid issues encountered with traditional screws. In our cohort of patients undergoing MIS SI joint fusion, clinical outcomes were favorable with 98% of patients experiencing a clinically significant benefit at 12 months. 

Postoperative complications were minimal. Two patients (5%) went on to have fusion surgery for significant degenerative disc disease or spinal stenosis. Both conditions were present prior to SI joint fusion surgery; however the patient's chief complaint was the SI joint necessitating primary attention to this area. There were 2 cases (5%) of transient trochanteric bursitis and 1 episode of piriformis syndrome. These are neither uncommon nor unexpected and can be a result of altered gait pattern due to low back or hip pain, postoperative hip abductor weakness, and other trauma in the region [19]. Facet pain was present in 20% of our patients, but it is unclear whether these patients had symptoms prior to the surgery. These patients were treated with either facet injections or physical therapy, depending on severity and patient preference. 

Although our study sample size is small, the results of minimally invasive SI joint surgery appear promising. All patients presented with low back and SI joint pain. Favorable outcomes in these patients underscore the necessity to suspect the SI joint as a pain generator in patients with low back pain. Special attention should be paid to the SI joint after lumbar spine surgery to avoid the potential for inaccurate diagnosis and treatment. Furthermore, this minimally invasive approach may significantly benefit the elderly population, who are not candidates for other conventional techniques due to poor bone quality, delayed healing and reduced mobility. Thirty-eight percent (38%) of our patients were over the age of 65. This segment of the population is not likely to respond well to physical therapy alone in part because of the degenerative nature of SI joint disease. The MIS procedure described herein may afford this segment of the population an opportunity to regain mobility, alleviate SI joint and low back pain caused by SI joint issues, and experience an improved quality of life.

## 5. Conclusion

When conservative measures fail, minimally invasive SI joint fusion using a series of triangular porous plasma coated titanium implants is a safe and effective treatment option in carefully selected patients. Additional prospective controlled trials are underway.

## Figures and Tables

**Table 1:** Patient demographics.

Patients	40
Age	58 (range 30–81)
Gender	30 F (75%), 10 M (25%)
Symptoms	39 (98%) LBP
	24 (60%) buttock pain
	5 (13%) groin pain
Prior lumbar spine surgery	19 (48%) total 12 fusion, 3 decompression, 1 unknown, 2 discectomy, 1 spinal cord stimulator

**Table 2:** Perioperative characteristics.

Joints treated	41
Right SI joint	17
Left SI joint	24
Concomitant spine procedures	1: L3/4 laminectomy, facetectomy, foraminotomy

**Table 3:** Postoperative complications and events.

Piriformis syndrome	1
New low back pain	1
Facet joint pain	8
Trochanteric bursitis	2
Discectomy at L4/5	1
Lumbar spine fusion	1 case at L2/3 due to severe spinal stenosis
	1 case at L3/4 for degenerative disc disease

## List of Abbreviations

SI: Sacroiliac
NRS: Numerical rating scale
MIS: Minimally invasive surgery
LBP: Low back pain.
